# Arterial Transit Time-corrected Renal Blood Flow Measurement with Pulsed Continuous Arterial Spin Labeling MR Imaging

**DOI:** 10.2463/mrms.mp.2015-0117

**Published:** 2016-05-09

**Authors:** Kazuhiro Shimizu, Nobuyuki Kosaka, Yasuhiro Fujiwara, Tsuyoshi Matsuda, Tatsuya Yamamoto, Tatsuro Tsuchida, Katsuki Tsuchiyama, Nobuyuki Oyama, Hirohiko Kimura

**Affiliations:** 1 Department of Radiology, Faculty of Medical Sciences, University of Fukui, 23-3 Matsuoka-Shimoaizuki, Eiheiji, Fukui 910-1193, Japan; 2 Radiological Center, University of Fukui Hospital; 3 Faculty of Life Sciences, Kumamoto University; 4 Global MR Applications and Workflow, GE Healthcare Japan Corporation; 5 Department of Urology, Faculty of Medical Sciences, University of Fukui

**Keywords:** arterial spin labeling, arterial transit time, magnetic resonance imaging, kidney, renal blood flow

## Abstract

**Purpose:**

The importance of arterial transit time (ATT) correction for arterial spin labeling MRI has been well debated in neuroimaging, but it has not been well evaluated in renal imaging. The purpose of this study was to evaluate the feasibility of pulsed continuous arterial spin labeling (pcASL) MRI with multiple post-labeling delay (PLD) acquisition for measuring ATT-corrected renal blood flow (ATC-RBF).

**Materials and Methods:**

A total of 14 volunteers were categorized into younger (n = 8; mean age, 27.0 years) and older groups (n = 6; 64.8 years). Images of pcASL were obtained at three different PLDs (0.5, 1.0, and 1.5 s), and ATC-RBF and ATT were calculated using a single-compartment model. To validate ATC-RBF, a comparative study of effective renal plasma flow (ERPF) measured by ^99m^Tc-MAG3 scintigraphy was performed. ATC-RBF was corrected by kidney volume (ATC-cRBF) for comparison with ERPF.

**Results:**

The younger group showed significantly higher ATC-RBF (157.68 ± 38.37 mL/min/100 g) and shorter ATT (961.33 ± 260.87 ms) than the older group (117.42 ± 24.03 mL/min/100 g and 1227.94 ± 226.51 ms, respectively; *P* < 0.05). A significant correlation was evident between ATC-cRBF and ERPF (*P* < 0.05, r = 0.47). With suboptimal single PLD (1.5 s) settings, there was no significant correlation between ERPF and kidney volume-corrected RBF calculated from single PLD data.

**Conclusion:**

Calculation of ATT and ATC-RBF by pcASL with multiple PLD was feasible in healthy volunteers, and differences in ATT and ATC-RBF were seen between the younger and older groups. Although ATT correction by multiple PLD acquisitions may not always be necessary for RBF quantification in the healthy subjects, the effect of ATT should be taken into account in renal ASL–MRI as debated in brain imaging.

## Introduction

Renal perfusion is one of the most important biological parameters for evaluating several renal diseases, including acute and chronic renal failure, kidney transplantation, and renovascular hypertension, as well as for pre-operative renal function assessment.^1–3^ For quantitative renal blood flow (RBF) measurement, nuclear medicine imaging using radiopharmaceuticals was regarded as the gold standard imaging technique and has, thus, been routinely performed in clinics for several decades.^2,4^ However, administration of radiopharmaceuticals caused internal radiation exposure, and its inherent poor spatial resolution limits detailed anatomical information. Furthermore, the high costs of radiopharmaceuticals increased medical costs. Therefore, frequent examinations were generally difficult in clinical circumstances. Recently, contrast-enhanced MRI has been successfully used to obtain quantitative RBF measurements to assess renal allograft nephropathy.^3^ However, due to the potential risk of nephrogenic systemic fibrosis, the use of gadolinium-based contrast agents has been limited in patients with impaired renal function.

Arterial spin labeling magnetic resonance imaging (ASL–MRI) is a non-contrast-enhanced perfusion-weighted MRI technique. It used magnetically-labeled water in blood as an endogenous tracer instead of externally-injected tracer, and it enabled non-invasive quantitative tissue blood flow measurements without ionizing radiation exposure and administration of contrast materials.^5^ This robust technique has now become routine in neuroimaging and is used for evaluating cerebrovascular disease, brain tumors, dementia, and other central nervous system (CNS) diseases.^6–8^ More recently, it has been applied to the kidney for several purposes, and a few study on the quantification of RBF for the evaluation of transplanted kidneys, renal artery stenosis, and renal tumors have been reported.^9–11^ Also it has been debated in neuroimaging, since arterial transit time (ATT) affected quantitative blood flow measurements in ASL–MRI, ATT correction is necessary for precise blood flow quantification.^5,12,13^ However, to the best of our knowledge, performance of ATT correction has been quite limited for renal imaging, and only a few studies have been reported so far; Cutajar et al. (2014) used a flow-sensitive alternating inversion recovery (FAIR) ASL with multiple inflow time (Tl) acquisitions and measured ATT-corrected RBF (ATC-RBF) for human kidneys.^14,15^ In this study, methods to measure both ATT and ATC-RBF were developed using pulsed continuous arterial spin labeling (pcASL) MRI with multiple post-labeling delay (PLD) acquisitions. Compared to FAIR–ASL, pcASL was theoretically expected to produce higher signal-to-noise ratio (SNR) images due to the longer temporal duration of the labeled bolus and higher labeled magnetization deliveries.^5^ The advantage of the pcASL technique may be that it allowed for accurate quantification, especially in lower RBF patients. For a proof of principle, this method was applied to two healthy subject groups, younger and older groups. Younger healthy subjects were generally expected to show faster ATT and higher RBF than older subjects.^16–18^ Therefore, we hypothesized that the pcASL with the multiple PLD methods would show this difference between the younger and older groups. Furthermore, to validate the RBF quantification, ATC-RBF measured by pcASL MRI was compared with effective renal plasma flow (ERPF) measured by ^99m^Tc-mercaptoacetyltriglycine (^99m^Tc-MAG3) renography, which was widely used to assess renal function clinically.

## Materials and Methods

### Subjects

This study was approved by the Institutional Ethics Committee. Informed consent was obtained from all subjects. A total of 14 healthy male volunteers were enrolled and categorized into younger (n = 8: age range 22–39 years, mean = 27.0 years) and older groups (n = 6: age range 53–75 years, mean = 64.8 years). Younger and older groups were defined as 20–40 years old and 50–80 years old, respectively. All volunteers had no history of renal disease, and their serum creatinine levels were measured before imaging to calculate the estimated glomerular filtration rate (eGFR). The eGFRs of all volunteers were normal: 92.09 ± 9.17 (range: 81.5–110.3) mL/min/1.73 m^2^ and 79.67 ± 11.45 (range: 62.3–95.8) mL/min/1.73 m^2^ for the younger and older groups, respectively. Blood pressures were 120.9/66.4 ± 9.0/12.0 (range: 104/52–133/89) mmHg and 124.7/76.3 ± 18.0/13.6 (range: 93/64–144/100) mmHg for the younger and older groups, respectively. All volunteers were fasting during the entire protocol period.

### pcASL MR protocols

MRI was performed using a 3.0-Tesla clinical scanner (Discovery MR750, GE Healthcare, Milwaukee, WI) with an 8-channel torso coil. The scout images were scanned with a gradient echo sequence in three planes through the center of each kidney. Coronal T_2_-weighted imaging covering the whole kidney was performed for anatomical and volume evaluation using a single shot fast spin echo (SSFSE) sequence with the following parameters: TR = 1123.2 ms, TE = 79.3 ms, image matrix 352 × 224, slice thickness: 5.0 mm, interval: 0 mm, flip angle: 90°, bandwidth: 83.33 kHz, and FOV = 38 × 38 cm.

ASL images were then acquired for quantitative measurements of ATT and ATC-RBF by applying pcASL with optimized background suppression and the 2D spin-echo echo planar imaging (EPI) sequence. The precise parameters and settings for pcASL were described in the previous study.^19^ The number of 180° pulses for background suppression was two, with each pulse applied for 1000 and 200 ms, respectively, before the beginning of EPI acquisition. The simulation confirmed that the first four slices were suppressed by <20%; using this condition, renal cysts were reasonably revealed as low non-perfusion areas (data not shown). For ASL imaging, multiple coronal slices covering the whole kidney were scanned with the following parameters: TR = 5500 ms, TE = 17.6 ms, image matrix: 96 × 128, slice thickness: 8 mm, interval: 0.5 mm, flip angle: 90°, bandwidth: 62.5 kHz, and FOV = 38 × 38 cm. Arterial labeling was performed with 2.0 s duration at the axial plane 10 cm superior to the center of the kidneys, and three different PLDs (0.5, 1.0, and 1.5 s) were set. A repetition time of 5.5 s was used to allow for recovery of the blood signal and for the subjects to breathe in during the quiet period between acquisitions, and a <17 s breath-hold was performed repeatedly at each TR for image acquisition. Nine averages of label and control were acquired for a total acquisition time of 3 min with each PLD setting. Measurement of the fully relaxed magnetization signal (reference images, *M*_0_) was also obtained to quantify RBF. The tag and control images acquired with ASL imaging were subtracted in a pairwise manner, and an averaged image was calculated using the script process on the MRI console to obtain perfusion images (Δ*M*).

### ATT and RBF calculation with the single-compartment model

**Fig 1.**
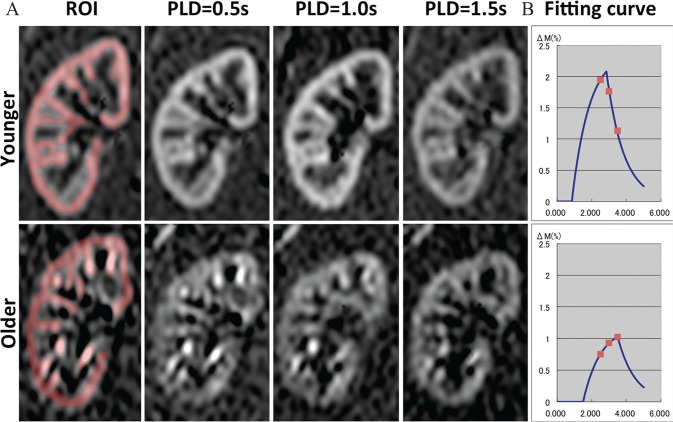
(**A**) Representative pulsed continuous arterial labeling (pcASL) images at the three different post-labeling delay (PLD) time points. Cortical signals in the younger group are visually stronger than in the older group. Representative cortical regions of interest (ROIs) are also shown in red. (**B**) Representative fitted % signal change curves of the single-compartment model. The signal peak of younger subjects is higher and sooner than that of older subjects.

All reference images (*M*_0_) and perfusion images (Δ*M*) acquired with multiple PLDs were transferred to the stand-alone workstation (iMac, OS X; Apple Computer, Cupertino, CA), and cortical ROIs were placed on the slices showing the renal hilum (4th slice from the front) using the image analysis software (OsiriX, version 5.6, http://www.osirix-viewer.com/index.html). Since the renal cortex showed a very high signal with good spatial resolution on ASL images, the cortical ROIs were drawn over the renal cortex on ASL images, and those ROIs were copied and pasted to the corresponding reference images. Samples of cortical ROIs are shown in Fig. 1A. Regarding the ASL signal model, since the tissue structure of the kidney consists of vascular-rich components in the renal cortices, we considered that the single compartment which mainly simulated the micro vascular signal would be appropriate for the kidney perfusion analysis. The measured cortical ROI values both from perfusion and reference images were then applied to the single-compartment model as described by the following formulae with the assumption that, by the time of image acquisition, all the labeled spins had left the vessel and resided in the parenchyma:

$$\begin{array}{ll} \Delta M(t) & =\frac{2\alpha f_{\text{r}}M_{0}}{\lambda }\cdot T_{\text{lapp}}\text{exp}\left(-\frac{\delta_{\text{a}}}{T_{\text{la}}}\right)\left[1-\text{exp}\left(-\frac{t-\delta_{\text{a}}}{T_{\text{lapp}}}\right)\right]\\ & \left(\delta_{\text{a}}<t\leq \delta_{\text{a}}+\tau \right)\\ \Delta M(t) & =\frac{2\alpha f_{r}M_{0}}{\lambda }\cdot T_{\text{lapp}}\text{exp}\left(-\frac{\delta_{\text{a}}}{T_{\text{la}}}\right)\left[1-\text{exp}\left(-\frac{\tau }{T_{\text{lapp}}}\right)\right]\\ & *\text{exp}\left[-\frac{t-(\delta_{\text{a}}+\tau )}{T_{\text{lapp}}}\right]\;(\delta_{\text{a}}+\tau \leq t)\\ 1/{\text{T}}_{\text{lapp}} & =1/T_{\text{ltissue}}+f/\lambda \end{array}$$

where *f*_r_ is RBF (i.e., ATC-RBF) measured with ASL imaging; *T*_1tissue_ is the tissue relaxation time of water (1150 ms was used for renal cortex)^20^; *λ* is the tissue blood partition coefficient of water; *τ* is labeling duration; and labeling efficiency (*α*) is assumed to be 0.75.^21^ *T*_1a_ is the arterial blood water relaxation time (assumed to be 1600 ms), and *δ*_a_ is the transit time the labeled spin took to travel from the tagging plane to the capillary bed (i.e., ATT). Using the above formulae, RBF and transit time were calculated under the condition of minimized sum of squares deviation from each data point and the model solution using the solver function of a spreadsheet program (Excel, Microsoft Corporation, Redmond, WA). In every case, it was confirmed that the optimized values were not extreme outliers by visual inspection on the graph of a simulated line and the acquired data points were automatically plotted on the same EXCEL sheet. Figure 1B is exactly the same graph as appeared on the sheet. When a fixed *δ*_a_ value was needed, it was only necessary to set the cell for *f*_r_ as the variable cell and run the solver tool. RBFs calculated from single PLD data sets were also obtained using the same single-compartment model assuming 1.0 s ATT. For comparison with ERPF, ATC-RBF and RBF were corrected by kidney volume (ATC-cRBF or cRBF), since ERPF was estimated on a per kidney basis. Kidney volumes (cm^3^) were calculated using image analysis software (Osirix) as follows: the area of the kidney on each T_2_-weighted coronal image covering the entire kidney was measured, and then the kidney areas were summed and multiplied by the slice thickness. Volume corrections were made with the following formulae: ATC-cRBF or cRBF = [ATC-RBF or RBF (mL/min/100 g)/100] × kidney volume (cm^3^).

### Renal scintigraphy

For ATC-RBF validation, renal dynamic scintigraphy was performed 1.5 h prior to the MRI scan using a clinical dual-head gamma camera (E.CAM, Siemens Healthcare, Erlangen, Germany) with a low-energy, high-resolution (LEHR) collimator. After the intravenous injection of 300 MBq of ^99m^Tc-MAG3 (FUJIFILM RI Pharma, Tokyo, Japan) in a supine position, serial images of 1.0 s per frame were obtained for the first 64 s, followed by 50 frames at 30 s per frame with a 128 × 128 matrix. Then, ROIs were placed over the left and right kidney each, and ERPF was calculated by a count-based gamma camera method^22^ using a commercially available nuclear medicine analysis system (Siemens ICON, Siemens Healthcare, Erlangen, Germany).

### Statistical analysis

All statistical analyses were performed using Graphpad Instat 3 (GraphPad Software Inc., La Jolla, CA, USA). Differences in ATT and ATC-RBF between groups were assessed by the Mann–Whitney test. The correlation between ERPF and ATC-cRBF or cRBF was tested by linear regression; *P* < 0.05 was considered significant.

## Results

**Fig 2.**
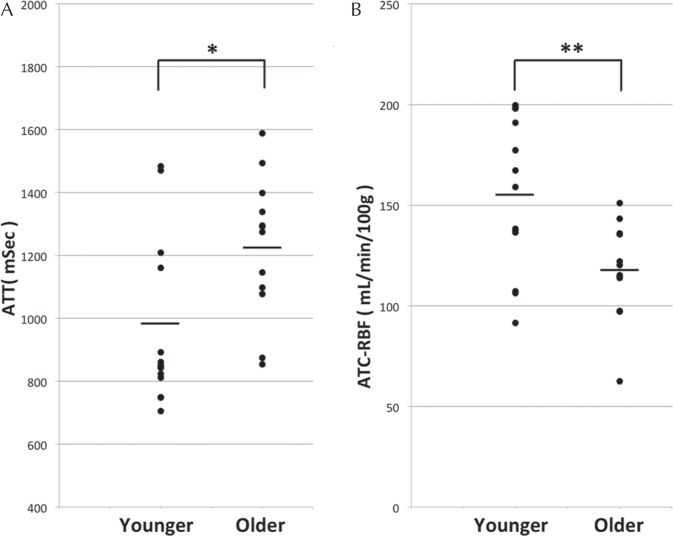
(**A**) Arterial transit time (ATT) and (**B**) arterial transit time-corrected renal blood flow (ATC-RBF) of the younger and older groups. The younger group shows significantly shorter ATT (961.33 ± 260.87 ms) and higher ATC-RBF (157.68 ± 38.37 mL/min/100 g) than the older group (1227.94 ± 226.51 ms and 117.42 ± 24.03 mL/min/100 g, respectively). *Bars* indicate the mean values of each group. *P < 0.001, **P < 0.05.

**Fig 3.**
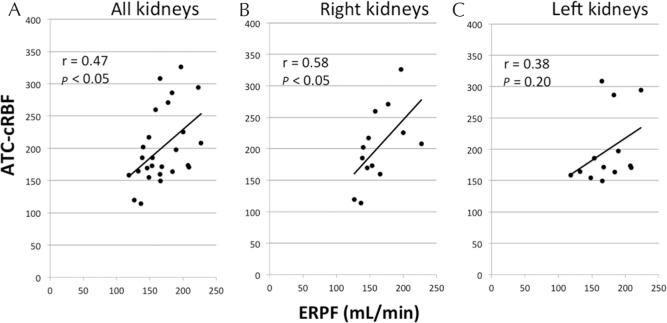
Scatter plots of kidney-volume and arterial transit time-corrected renal blood flow (ATC-cRBF) and effective renal plasma flows (ERPF) of (**A**) all the kidneys, (**B**) right kidneys, and (**C**) left kidneys. Significant correlations between ATC-cRBF and ERPF are identified for all the kidneys and right kidneys, while no significant correlation was seen for left kidneys.

All image acquisitions and post-processings were successful, except in one young, physically lean subject due to extreme kidney image distortion. The technical success rate was 92%. Representative pcASL images at different PLD time points and the % signal change curves of the compartment analysis of younger and older subjects are shown in Fig. 1. Signal intensities and peaks were generally stronger and faster in the younger volunteers than in the older volunteers. The mean ATC-RBF of the renal cortex of all subjects was 139.10 ± 37.93 mL/min/100 g. The younger group had significantly higher ATC-RBF (157.68 ± 38.37 mL/min/100 g) and shorter ATT (961.33 ± 260.87 ms) than the older group (117.42 ± 24.03 mL/min/100 g and 1227.94 ± 226.51 ms, respectively) (Fig. 2). A significant linear correlation was seen between ATC-cRBF and ERPF in all kidneys (r = 0.47, *P* < 0.05) (Fig. 3A). When the left and right kidneys were analyzed separately, a stronger linear correlation was observed for the right kidney (r = 0.58, *P* < 0.05), while no significant correlation was seen for the left kidney (r = 0.38, *P* = 0.20). (Fig. 3B, C)

**Fig 4.**
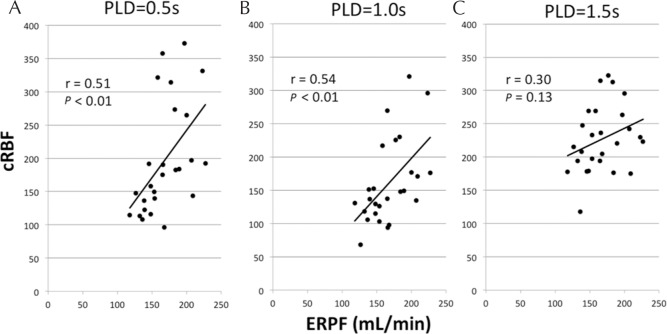
Scatter plots of effective renal plasma flow (ERPF) and kidney volume-corrected renal blood flow (cRBF) calculated from single post-labeling delay (PLD) data of (**A**) 0.5 s, (**B**) 1.0 s, and (**C**) 1.5 s. The cRBFs from 0.5 and 1.0 s single PLD data showed significant linear correlations to ERPF, while the cRBF from 1.5 s single PLD data showed no significant correlation.

To compare the multi-PLD and single-PLD methods, cRBF with single-PLD methods (i.e., no ATC correction) was calculated assuming 1.0 s ATT from the same data sets. As shown in Fig. 4, single PLD acquisitions with 0.5 and 1.0 s PLDs showed significant linear correlations with ERPFs that were comparable to those with multiple PLD acquisitions. However, when a relatively long PLD (1.5 s) was used for the single PLD acquisitions, there was no significant correlation between ERPF and cRBF measured by single PLD acquisition.

## Discussion

This study demonstrated the feasibility of ATT and ATT-corrected RBF measurements using pcASL with multiple PLD acquisition in healthy subjects. ATT correction of ASL–MRI for human kidney imaging has not been well evaluated so far, although it has been well debated in neuroimaging. To the best of our knowledge, few studies performing ATT correction for human kidney imaging by the FAIR–ASL technique have been reported,^14,15^ and ATT itself has not yet been measured for human kidney. Regarding ATT in the brain, age-related ATT prolongation has been reported, with the younger group showing significantly shorter ATTs in cerebral gray matter than the elderly group.^16,17^ Moreover, ATTs obtained with multiple PLD ASL–MRI change dramatically in patients with chronic occlusive cerebrovascular disease.^23^ Such ATT differences were well known to affect CBF quantification in the ASL signal model, because it assumed that all tagged signals had reached the acquisition plane.^13^ When the timing of ASL signal acquisition (i.e., PLD) was earlier or later than the ATT, the regional ASL signal should diminish, resulting in CBF underestimation with ASL–MRI. To compensate for this problem, ATT correction with multiple PLD acquisitions was regarded as an essential process for precise CBF quantification in the brain ASL–MRI model.^12^ In this study, age-related prolongation of ATT also occurred in renal ASL–MRI. Moreover, the ATC-cRBF showed a significant correlation to ERPF, but the cRBF with suboptimal single PLD settings resulted in a poorer correlation to ERPF. In the renal cortex, afferent arterioles branching from interlobular arteries form the glomerulus and then drain directly to efferent arterioles; they then finally flow into the peritubular capillary network located mainly in the renal medulla.^24^ Moreover, renal arteries branch directly from the aorta with high pressure, and they were not tortuous like the internal carotid and vertebral arteries. Therefore, inflows and outflows of labeled water in renal cortex may be faster than in brain tissue where labeled water distributed into interstitial tissues. In such circumstances, acquisition timing with a suboptimally long PLD, such as 1.5 s, may be too late in some cases, and it could cause underestimation of RBF. On the other hand, single PLD acquisitions with PLD = 0.5 and 1.0 s showed comparable correlations to ERPF compared to multiple PLD acquisitions, because these PLDs may be appropriate acquisition timing to represent comparable % signal change curves fitted by multiple PLD data. Therefore, in the healthy populations tested in this study, ATT correction by multiple PLD acquisitions may not always be necessary for RBF quantification. However, the situation may be more complicated for clinical application. Thus, the actual ATT and optimal PLD may vary for each patient, resulting in suboptimal quantification of RBF. In such situations, ATT correction by multiple PLD acquisitions could facilitate precise RBF quantification by ASL–MRI. Moreover, the present method enabled ATT measurement, which was difficult to accomplish by other imaging methods. The utility of ATT itself for understanding the pathophysiologic status of cerebrovascular disease, as well as of CBF, has been reported.^23,25^ Likewise, renal ATT had a potential to provide additional information for assessment of renovascular disease, where conventional MRI can only contribute to assessing the morphological changes at present.

The correlation between ATC-cRBF and ERPF was significant, but more modest than expected. Such modest correlations of RBF measured by ASL–MRI against gold standards have been reported. For instance, Ritt et al. (2010) reported a similar modest correlation between para-aminohippuric acid (PAH) plasma clearance and FAIR–ASL in 24 metabolic syndrome patients,^26^ while Wu et al. (2011) also reported a moderate correlation between pcASL–MRI and dynamic contrast-enhanced MRI in 19 healthy subjects.^27^ One limitation of this study was that only healthy subjects were recruited, so that the range of renal function was relatively narrow. This may partly explain the modest correlation observed in this study. Another concern was that use of a tubular secretion tracer, such as MAG3 and PAH, may not always provide renal plasma flow, because clearance of such tracers was determined not only by renal plasma flow, but also by tubular secretory function.^4^ Thus, ERPF does not represent true renal plasma flow in a subject with renal tubular dysfunction. Furthermore, the pharmacokinetics of MAG3 differed from those of PAH or radioiodine-labeled hippurate (OIH, analogue of PAH), whose plasma clearances are regarded to be a good standard for renal plasma flow. MAG3 showed higher protein binding, slower blood clearance, higher extraction efficiency by tubular cells, and larger excretion into the bile than OIH and PAH.^28^ However, all subjects enrolled in this study were healthy, without any history of renal disease. In such populations, plasma clearance of MAG3 showed an excellent correlation with that of OIH; thus, MAG3 is now widely used to evaluate renal function in clinics as an alternative to OIH.^28,29^ In addition, since only linear correlations between ERPF and ATC-cRBF, not absolute values of renal plasma flow were evaluated in this study, this may not have been a critical problem.

It was more likely that technical limitations of ASL–MRI may explain such a modest correlation between ATC-cRBF and ERPF, including the susceptibility effect around the kidneys, effects of pulsation and susceptibility at the labeling plane, and misregistration due to respiratory motion. As shown in the results, the left kidney showed a poorer correlation between ATC-cRBF and ERPF than the right kidney. The reason for this difference was still unknown, but one possible explanation was that acquisition of the ASL signal from the left kidney may be more hampered by susceptibility effects compared to the right kidney, because the left kidney was generally located closer to air in the stomach and lungs, which caused an inhomogeneous magnetic field. In this study, in particular, the 2D-EPI readout sequence was used in ASL–MRI, which was the most efficient usage of MR signal available per unit time, but it was more sensitive to susceptibility effects. Other readout sequences, such as fast spin echo (FSE) or balanced steady-state free precession (SSFP) sequence, may be more suitable under such circumstances,^30^ because they were less sensitive to susceptibility effects. However, their lower SNR in ASL–MRI will be a trade-off compared to the EPI readout sequence. The second limitation of renal ASL was labeling efficiency at the labeling plane. For renal pcASL–MRI, spin labeling took place at the aorta around the diaphragm, where air in the lungs caused strong susceptibility effects; thus, labeling efficiency may vary depending on individuals. Moreover, unstable ASL tagging due to cardiac pulsation and the effects of flow dispersion have been reported in neuroimaging,^31,32^ which may be more problematic in stronger pulsatile blood flows within the aorta. In fact, the peak flow velocity in the aorta may outrange the supposed flow range for CNS ASL imaging from the result of the simulated efficiency of pcASL.^19^ Such effects may have affected RBF quantification in this study.

Another big issue for renal ASL–MRI was how to deal with respiratory movements during acquisition. In this study, voluntary synchronized breathing, with multiple sessions of ∼17 s breath-holds, was used, and the data were summed. Although no problem was seen, it may potentially cause blurring and misregistration, which have some effect on RBF quantification. For other approaches, Robson et al. (2009) reported that retrospective image sorting improved image quality with free breath acquisition,^21^ while Tan et al. (2014) reported the feasibility of respiratory navigator-gated acquisition.^33^ Such “subject-independent” techniques may be needed for clinical applications in the future, since many patients have difficulties with appropriate respiratory control. In addition, this may be another advantage of renal ASL. In this study, only three PLD time points were obtained to remain within an acceptable scanning time and decrease subjects’ physical burden. However, more PLD time points would be desirable for more precise ATT and RBF measurements. Recently, brain ATT-corrected pcASL–MRI with low-resolution multiple PLD acquisitions has been reported.^34^ This method enabled more PLD-time points to be obtained without elongating scanning time, leading to more precise ATT and ATT-corrected cerebral blood perfusion measurements. Combined with the development of free-breathing acquisition, such a technique could also be applied to renal ASL–MRI in the future.

## Conclusion

Calculations of ATT and ATC-RBF by pcASL with multiple PLD were feasible in healthy volunteers. Even in healthy subjects, differences in ATT, as well as ATC-RBF, were seen between younger and older groups. Although ATT correction by multiple PLD acquisitions may not always be necessary for RBF quantification in the healthy subjects, the effect of ATT should be taken into account in renal ASL–MRI as debated in brain imaging. However, the significant but modest correlation between ATC-cRBF and ERPF observed in this study suggested that further technical development may be needed for more precise RBF quantification by pcASL–MRI.
